# Efficacy of Perceptual Learning-Based Vision Training as an Adjuvant to Occlusion Therapy in the Management of Amblyopia: A Pilot Study

**DOI:** 10.3390/vision5010015

**Published:** 2021-03-23

**Authors:** Carlos Javier Hernández-Rodríguez, Hideki Fukumitsu, Pedro Ruiz-Fortes, Roberto Soto-Negro, María Merino-Suárez, David P. Piñero

**Affiliations:** 1 Department of Ophthalmology, Vithas Medimar International Hospital, 03016 Alicante, Spain; hernandezrodriguezcj@gmail.com (C.J.H.-R.); hideki.fukumitsu@gmail.com (H.F.); RuizFP@vithas.es (P.R.-F.); SotoNR@vithas.es (R.S.-N.); 2 Group of Optics and Visual Perception, Department of Optics, Pharmacology and Anatomy, University of Alicante, 03690 Alicante, Spain; 3 Department of Ophthalmology, Marina Baixa Hospital, 03570 Alicante, Spain; maria_merino82@hotmail.com

**Keywords:** amblyopia, perceptual learning, occlusion therapy, patching, vision therapy

## Abstract

A retrospective study was conducted to evaluate preliminarily the efficacy of perceptual learning (PL) visual training in medium-term follow-up with a specific software (Amblyopia iNET, Home Therapy Systems Inc., Gold Canyon, AZ, USA) for visual acuity (VA) and contrast sensitivity (CS) recovering in a sample of 14 moderate to severe amblyopic subjects with a previously unsuccessful outcome or failure with patching (PL Group). This efficacy was compared with that achieved in a patching control group (13 subjects, Patching 2). At one-month follow-up, a significant VA improvement in the amblyopic eye (AE) was observed in both groups, with no significant differences between them. Additionally, CS was measured in PL Group and exhibited a significant improvement in the AE one month after the beginning of treatment for 3, 6, 12, and 18 cycles/º (*p* = 0.003). Both groups showed long-lasting retention of visual improvements. A combined therapy of PL-based visual training and patching seems to be effective for improving VA in children with amblyopia who did not recover vision with patching alone or had a poor patching compliance. This preliminary outcome should be confirmed in future clinical trials.

## 1. Introduction

Amblyopia is a neurodevelopmental visual disorder that emerges because of a binocular disturbance in the earlier years due to anisometropia, strabismus, or both, and occasionally due to pathology. Amblyopic subjects show a reduced visual acuity (VA) and contrast sensitivity (CS) in one eye, rarely both, and it is accompanied by decreased or null stereopsis and a wide array of visual deficits, such as fixation instability, and perceptual, accommodative, and oculomotor disorders [1,2,3]. Amblyopia is typically treated with an optimal refractive prescription and patching. At present, occlusion therapy is widely known and remains being the gold standard for recovering the VA in patients with amblyopia. However, there are patients who do not have a good response with patching, some due to a poor compliance [4] and others due to some sort of patching resistance. One of the patching resistance causes may be the consequence of the negative effect that occlusion has on binocularity. Narasimhan et al. [5] reported the crucial role that suppression plays in amblyopia and its likely relation with patching-resistant patients. Furthermore, Hess et al. [6] observed that after short-time occlusion, when the patch is removed, the previously patched eye temporally increases its contribution to binocular vision due to a stronger signal from that eye. Therefore, they suggested that occlusion of the FE improves VA of the AE but also could increase interocular suppression. This fact is controversial and is still not well understood but may explain why some patients do not improve with treatment and encourages clinicians to seek for alternatives which can act as an adjuvant to occlusion.

In the last years, technological progress and the advance in the knowledge about neural mechanisms of amblyopia had allowed researchers and clinicians to develop new therapeutic approaches based on vision training using specific visual stimuli promoting visual improvement due to neuroplasticity [7], and even involving attentional skills using video games [8]. This is the case of vision therapy using perceptual learning (PL) techniques, which consists of the stimulation of the amblyopic eye through different repeated visual tasks [9]. In PL training sessions, Gabor’s patches, letter optotypes, or random-dot stimuli are normally presented in digital devices, with the patient being required to do different tasks such as grating orientation discrimination or letter recognition, among others [9,10]. PL is commonly used to increase VA and CS, but can transfer its improvement to other visual tasks (e.g., foveal crowding or Vernier acuity) [11]. PL training can be performed monocularly, with a patch in the fellow eye (FE) [12,13], or as a part of dichoptic training (DT), where patients use polarized or red-green glasses to do binocular tasks [14,15], or even virtual reality head-mounted displays (VR-HMD) [16,17]. Moreover, some authors even reported interesting and positive results combining PL-based therapy with different kind of non-invasive transcranial stimulation [18,19]. 

There are some aspects about the use of PL training in amblyopia that need more discussion and research. First, dose–response ratio in amblyopia treatment seems to be better with PL training or when combining PL with patching than only using patching [14]. Likewise, it should be noted that amblyopic patients are usually children and the use of videogames for PL training helps children to maintain attention in visual tasks and might improve compliance treatment [20]. Finally, there is scientific evidence demonstrating the efficacy of the application of perceptual learning for the treatment of amblyopia not only in children [14,21,22,23], but also in patients beyond the critical period, as adults [24,25,26]. It is important to highlight the relevance of selecting appropriate stimuli and visual tasks for an adequate stimulation of the amblyopic eye (AE), such as Gabor patches, letter optotypes, Vernier´s stimuli, or random-dot stereograms that have been proven to provide the cortical stimulation required to improve the eye resolution [27]. In this study, the Amblyopia iNET software was investigated as it uses letter optotypes in different tasks of orientation identification, visual memory, and tracking in crowding environments as a part of short videogames for training the VA and CS of the AE. VA and CS are visual parameters directly related to the function of primary visual cortex (V1), which is one of the main brain areas affected by amblyopia [28]. 

According to a recent systematic review, there are some aspects that should be considered in the investigation of new vision therapy treatments for amblyopia [29]. Frequently, published articles do not clearly short by type of amblyopia and it can be a source of bias due to cortical differences between anisometropic and strabismic amblyopia [30,31]. Furthermore, there is a need of outcomes for longer follow-up periods comparing with a control or placebo group. Therefore, the main purpose of the current retrospective study was to assess the efficacy of perceptual learning training with a specific software (Amblyopia Inet, Home Therapy Systems Inc., Gold Canyon, AZ, USA) for VA and CS recovering in a sample of moderate to severe amblyopic subjects comparing with a patching control group in medium-term follow-up. 

## 2. Materials and Methods 

### 2.1. Patients

This is a retrospective study based on clinical records of amblyopic children treated in the Department of Ophthalmology of the Medimar International Hospital (Alicante, Spain). The sample evaluated was divided into two groups depending on the type of treatment: PL Group (n= 14, 6 males and 8 females, mean age: 9.7 ± 1.4 years) including subjects in which the combination of spectacle correction, patching, and visual training using a PL environment was prescribed (Amblyopia iNET, Home Therapy Systems Inc., Gold Canyon, USA), and Patching Group (n = 13, 6 males and 7 females, mean age: 5.0 ± 0.4 years) including subjects treated with spectacle correction and patching. Inclusion criteria for the study were the following:

- Subjects until 17 years old with moderate or severe amblyopia.
- Anisometropic amblyopia (differences in sphere between eyes of more than 1.0 D and in cylinder of more than 1.5 D) or strabismic amblyopia (any angle of deviation).
- No active ocular or systemic disease.
- No previous ocular surgery.

The research adhered to the principles of the Declaration of Helsinki and was approved by the ethics committee for medical research of the Health Department of Alicante (General Hospital, Alicante, Spain) (CEIm 2020-061, ISABIAL 200149).

### 2.2. Clinical Protocol

In all cases, children had undergone a complete baseline examination including measurement of uncorrected and corrected visual acuity with Snellen chart, manifest refraction with and without cycloplegia, cover test, Worth 4-dot test, stereopsis measurement (TNO stereo-test), 4 prism diopter base-out prism to check the presence or not of microtropia, and ocular motility examination. In all cases, spectacle correction was prescribed, with a rechecking at 2 to 4 months after. In this recheck visit, patching was recommended following the PEDIG (Pediatric Eye Disease Investigator Group) guidelines [32]. Children not improving with patching after two consecutive follow-up visits or with a low level of compliance were derived to the Vision Therapy Unit and a PL-based visual training was initiated. In PL group, changes in photopic contrast sensitivity were also analyzed for 3, 6, 12 and 18 cycles/degree using the CSV-1000 test (VectorVision, Greenville, OH, USA) which is based on two forced-choice answers. Children underwent PL-based therapy until amblyopia resolution or no improvement in two consecutive visits (i.e., three months of treatment). Regular exams of control of VA and refraction were performed in the hospital at 1, 3, 6, 9, 12, 15, and 18 months after initiating the combined treatment. Likewise, in the Patching Group, changes in CS were also evaluated during the follow-up. Improvement is indicated by lower scores of VA measured with Snellen chart and higher scores of CS measured with the CSV-1000 test during follow-up. Finally, compliance of the PL therapy in the first two follow up checks, one and three months, was assessed as the parentage of the training time performed and the estimated.

### 2.3. Perceptual Learning Software

**Figure 1:**
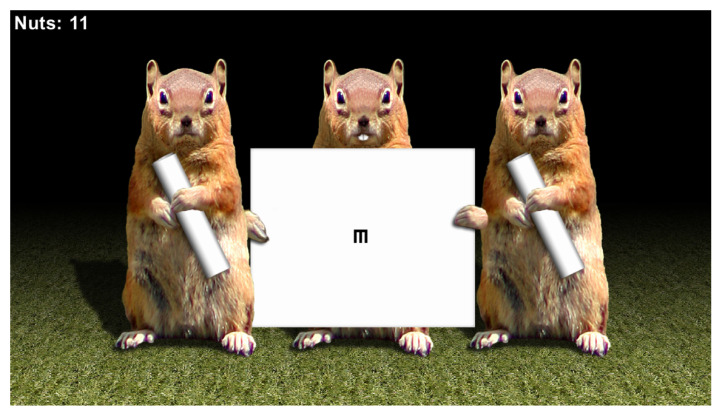
Game called “Chipmunk chase” based on orientation discrimination with Snellen E optotypes.

**Figure 2:**
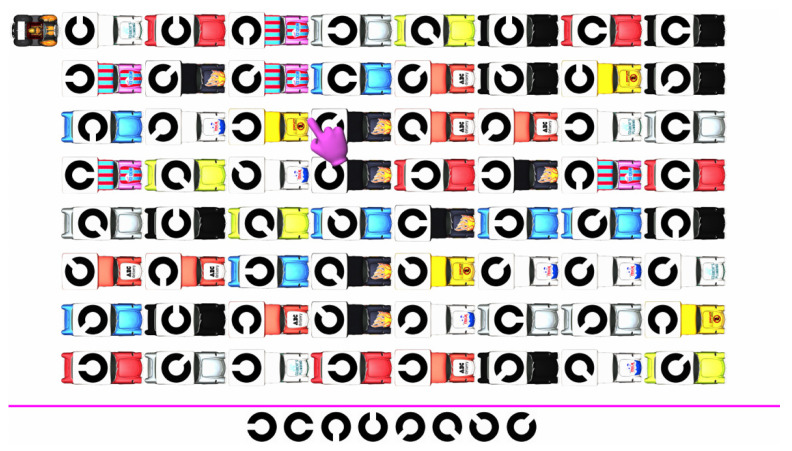
Game called “Traffic jam” based on orientation discrimination with Landolt C optotypes.

Amblyopia iNET is a perceptual learning software that is installed in the personal computer of the patient for home training. The visual training consisted of sessions of 30 min performed at home five days per week with the dominant eye occluded and maintaining a distance from the monitor of around 40 cm. These sessions were controlled online by the optometrist, confirming the level of compliance and customizing sessions selecting among the different games available. In addition, notes or instructions can be sent to patients through the software. Before training, there was a test for calibrating the device according to the size and resolution of the screen. Afterwards, in each training session, the patient performs letter recognition, orientation identification, and tracking in crowding environments, with specific sounds associated with right and wrong answers to provide a feedback to patients and to maintain attention; all tasks used either high contrast Landolt Cs, Snellen Es or other symbols. Visual demands increased by changing the size, speed of presentation, and level of crowding when the patient levels up after obtaining less than 20% of incorrect answers. During the follow-up, the practitioner can check the results, analyzing the percentage of correct answers, the estimated VA according to the stimuli parameters during tasks, the level of difficulty, and time and date of the session. Exercises consist of the following child-friendly short video games:

- Follow the letter: Orientation identification with a moving Landolt C of increasing resolution.
- Letter jump: Orientation identification with a jumping Landolt C of increasing resolution that appears in different parts of the screen.
- Find the target: Letter discrimination using a reference letter or symbol that must be found by the patient among many crowded letters or symbols.
- Concentration: Optotype or symbol identification in a list of stimuli after its previous presentation during a short period of time.
- Capture the target: Identification of an optotype or symbol among a list of optotypes and symbols that are moving constantly across the screen.
- Space ball: Eye-hand coordination exercise in which a space ball must be kept on the field of the game with a moving stimulus (a planet or similar) and 4 paddles, one of each side of the screen. The patient should move a paddle to bounce the ball away from the side. The patient will use the mouse to move the bars (up, down, right, and left of the screen) to hit the ball and prevent it from exiting from the screen.
- Chipmunk chase: Orientation discrimination with Snellen Es presented by 3 squirrels (Figure 1).
- Penguin peek: Orientation discrimination in several penguins holding a card with stimuli (Forms, E or C). For each penguin, there will be another penguin holding a card with the stimulus partner. Using the mouse, the patient will click on a penguin and then on another penguin that the stimulus partner is holding.
- Skiing: Eye-hand coordination with a mouse; the patient must pass between a pair of moving poles a small penguin that can be moved across the screen.
- Traffic jam: Orientation discrimination of Landolt C optotypes printed on the roof of several crowded colored and noisy cars (Figure 2).
- Laser ball: Orientation discrimination of a Landolt C of increasing resolution. The patient will use the mouse to move the laser ball to the end of the screen and find the stimulus in the upper half of the screen that matches that of the laser ball. The patient will move the mouse directly under the corresponding stimulus, and then will press the mouse button to launch the laser ball.

### 2.4. Data Analysis

Data were analyzed using the software SPSS Statistics v.24 (IBM, Armonk, NY, USA). The following variables were considered in the analysis: type of amblyopia, previous treatment, and patching regimen. Non-parametric tests were used as most of data samples were not normally distributed (Kolmogorov–Smirnov test). Mann–Whitney U test was used for comparison of independent paired samples, Wilcoxon signed-rank test was used for comparison of dependent paired samples, Kruskal–Wallis test was used for the comparison of multiple independent samples, and Spearman´s rho was used for analyzing the level of correlation between different clinical variables. A two-tailed *p*-value < 0.05 was considered as statistically significant. In addition, Cohen´s d was calculated to obtain the effect size of the treatments and a comparison between them. 

## 3. Results

### 3.1. Description of the Sample

Descriptive characteristics of the subjects included in each group in the current study are summarized in Table 1. There were significant differences between groups in age and the rate of previous treatments. Subjects in the PL Group were significantly older than subjects in the Patching Group (*p* = 0.006). Regarding previous treatment, 12 out of 14 subjects of the PL Group received previous patching treatment, against 3 out of 13 subjects in the Patching Group (*p* = 0.001). Additionally, two patients with eccentric fixation and two patients with eccentric fixation and anomalous correspondence were included in the PL Group. There were not significant baseline differences between groups in gender (*p* = 0.863), type of amblyopia (*p* = 0.842), and patching regimen (*p* = 0.440). Concerning the baseline visual examination, no significant differences between groups were found in the sphere (*p* = 0.342) and cylinder (*p* = 0.228) of the FE, sphere (*p* = 0.752) and cylinder (*p* = 0.338) of the AE, and baseline VA of the FE (*p* = 0.519) and AE (*p* = 0.350).

### 3.2. Changes in Visual Acuity

**Figure 3:**
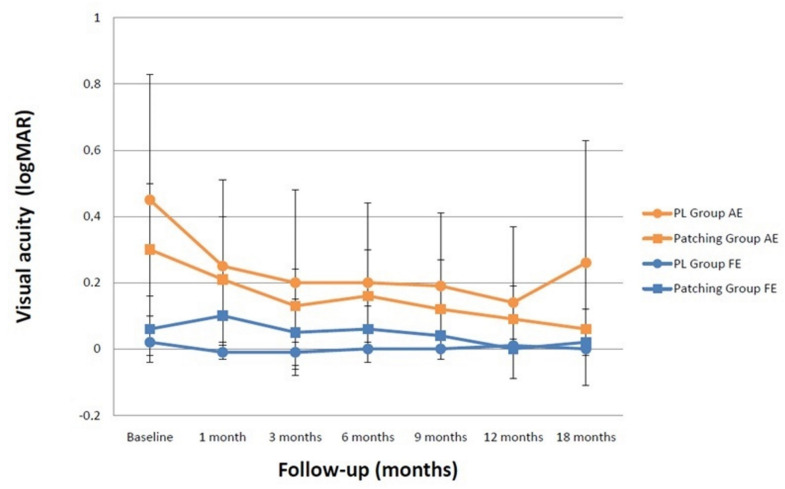
Visual acuity of perceptual learning group (PL Group) and patching group. Blue lines are for fellow eyes (FE) and orange lines for amblyopic eyes (AE).

Changes in VA during treatment with comparison between groups are displayed in Table 2. At one month follow-up, results showed a significant VA improvement in the AE from 0.45 ± 0.38 logMAR to 0.25 ±0.26 logMAR (Z = −3.182, *p* = 0.001) in the PL Group and from 0.30 ± 0.20 logMAR to 0.21 ± 0.19 in the Patching Group (Z = −2.366, *p* = 0.018), with no significant differences between groups (Z = −0.159, *p* = 0.877) (Figure 3). The enhancement in VA was 0.22 ± 0.13 logMAR in the PL Group and 0.14 ± 0.10 logMAR in the Patching Group, with the PL Group showing a slightly non-significant higher increase in VA after one month (*p* = 0.874). Furthermore, there were not differences by type of amblyopia in the PL Group (*p* = 0.071) and the Patching Group (*p* = 0.077). VA was slightly better in the Patching Group, but with no significant differences between groups at 3, 6, 9, 12, and 18 months follow-up (*p* > 0.05). At one month follow-up, the effect size with Cohen´s d was 0.61 for PL and 0.46 for patching, and 0.17 when comparing both treatments. Furthermore, there were no regressions in the visual outcome achieved in both groups. It should be pointed out that at 18 months after treatment, VA of AE in the PL Group was worse compared to the Patching Group, although the difference did not reach statistical significance. This is probably because a nine-year-old subject from the PL Group, who presented a severe amblyopia with VA of 1.30 logMAR, did not use neither the optical prescription, nor patch until the beginning of the study. In addition, he did not adequately undergo the vision therapy treatment; therefore, his VA was around 0.82 logMAR during the follow-up. 

Additionally, a sub-sample of six subjects who were included in the vision therapy group due to non-compliance of patching showed similar results with PL-based therapy and no occlusion than the whole group. Specifically, the baseline VA of AE experienced a significant improvement from 0.41 ± 0.45 logMAR to 0.20 ± 0.30 logMAR (Z = −2.201, *p* = 0.028). In the FE, there was a marginal and no significant improvement of VA in both groups (*p* > 0.05). In addition, a minimal but statistically significant difference between groups in the VA of the FE was found at one month of follow-up, with no significant differences during the rest of the follow-up (*p* > 0.05).

### 3.3. Changes in Contrast Sensitivity in the Perceptual Learning Group and Correlation with Visual Acuity Outcomes

**Figure 4:**
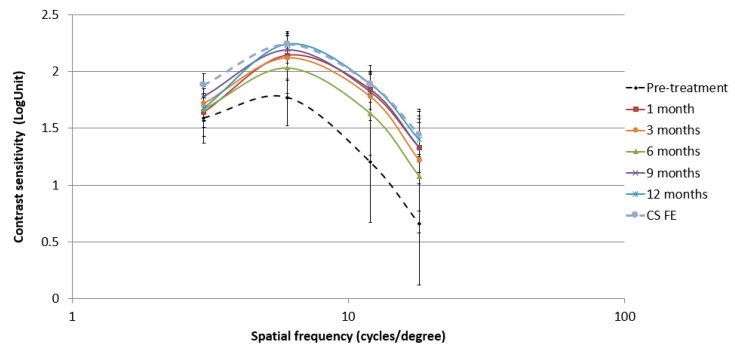
Changes in contrast sensitivity (CS) in the amblyopic eye during the follow-up compared to the baseline values of CS in the fellow eye (FE).

A significant improvement in CS of the AE was observed in PL Group one month after the beginning of treatment for 3 (*p* = 0.036), 6 (Z = −2.791, *p* = 0.005), 12 (Z = −2.941, *p* = 0.003) and 18 cycles/º (Z = −2.943, *p* = 0.003). The increment in log units of CS at 1-month visit was 0.17 ± 0.23, 0.30 ± 0.24, 0.44 ± 0.32, and 0.51 ± 0.38 for 3, 6, 12, and 18 cycles/º, respectively, and the effect size was 0.23, 1.60, 1.59, and 1.51 for 3, 6, 12, and 18 cycles/º, respectively. This improvement in CS tended to increase slightly until the end of the monitoring period, but with no significant differences for any spatial frequency during the rest of the follow-up (*p* > 0.05). Figure 4 shows the change in CS of the AE in the vision therapy group. No significant differences in CS of the FE were observed for any spatial frequency comparing pre-treatment and one-month follow-up values (*p* > 0.05). In addition, there was a significant and strong correlation between the change in VA and CS for 18 cycles/º at one month after starting the treatment (r = −0.82, *p* = 0.002). 

The comparison of CS of AE and FE in PL Group reveals the presence of statistically significant differences for 3 (*p* = 0.005), 6 (*p* = 0.002), 12 (*p* = 0.003), and 18 cycles/º (*p* = 0.005), confirming that the AE had worse CS for all spatial frequencies. At one month after initiating the treatment, FE and AE showed similar CS values for 3 (*p* = 0.655), 6 (*p* = 0.999), 12 (*p* = 0.655), and 18 cycles/º (*p* = 0.317). This trend was maintained during the remaining follow-up.

### 3.4. Compliance and Duration of Perceptual Learning Therapy

The treatment time was variable among subjects, but all of them performed at least three months of PL therapy, except in one case who did not want to continue and left the treatment after one month of training. According to the duration and number of sessions, 11 h of PL training were estimated for the one-month follow-up check, and 33 h for the three-month follow-up check. The mean training time performed by patients was 4.42 ± 1.84 h (from 1.5 to 7.5 h) in the first month, and 9.46 ± 5.64 (from 1.5 to 20.5 h) at three months. Thus, the compliance was around the 40.2% at one month and decreases to 30.7% at three months.

## 4. Discussion

In this study, the VA of AE improved with patching and vision therapy (combined in most of cases with patching) treatment one month after starting the study and was stable until the end of the follow-up. This enhancement was similar for both types of treatments, with an effect size of 0.17 when comparing treatments, although it was higher for PL (d = 0.61) than patching (d = 0.46) when comparing pre-post results at one month follow-up. It means that PL seems to cause a larger impact on VA than patching in this sample, even though, the Patching Group had a slightly better VA than PL Group at the end of follow up. However, it should be pointed out that there were significant differences in baseline characteristics between groups that should be considered. On the one hand, patients included in PL Group did not experience previously an improvement in VA with patching due in many cases to very poor compliance, and therefore patching alone was not enough to improve VA before vision therapy. Furthermore, baseline VA of subjects in PL Group tended to be worse, although the difference with the Patching Group did not reach statistical significance. On the other hand, subjects in PL Group were significantly older than subjects in Patching Group. Both characteristics, patching compliance and age, are potential limitations for achieving an adequate rehabilitation according to scientific literature [4,33]. Furthermore, the sub-sample of patients in PL Group with mild amblyopia who did not undergo patching at all also showed an improvement in VA. Therefore, PL may be an option to promote VA recovery in those cases in which patching is not enough for achieving a successful visual outcome or the compliance is very poor. Additionally, in this study, there were not significant differences in VA between patients with and without strabismus during follow-up, and therefore PL may be beneficial for both types of amblyopia. Nonetheless, results should be investigated further in future clinical trials including large sample sizes, sorting results according to the type of amblyopia to avoid the probable source of bias of mixing anisometropic and strabismic amblyopic cases due to the cortical differences between the two types of amblyopia.

The improvement observed in VA of patients treated with spectacle correction, patching in most of cases and PL-based vision therapy was consistent with that reported by others authors. Gambacorta et al. [14] reported an improvement of 0.1 ± 0.003 logMAR after 10 h of PL, Cheng et al. [25] reported an improvement of 1.64 ± 0.06 lines, and Deshpande et al. [22] showed a VA increase of three lines with PL training in anisometropic amblyopia, with the highest change occurring between the third and fourth week of treatment. Additionally, Avram et al. [13] also reported a significant VA improvement using Amblyopia iNET in a small case series, obtaining VA decimal values of 0.8 or more after treatment. Furthermore, Deshpande et al. [22] and Zhang et al. [12] also described that the increase of VA is similar with PL-based visual training and patching. However, Lee et al. [34] reported no VA improvement after PL training in patching resistant patients, but it was for binocular summation and reading speed. Discrepance between Lee et al. results and other studies could be because of methodological differences, such as the sample size and clinical protocols.

Concerning CS, it was measured in the PL Group to confirm if PL-based vision training helped to increase its baseline values, as reported by other authors also using PL training [13,25,35]. After one month of treatment, a significant improvement in CS for all spatial frequencies was found in the current series, with a higher increase for the spatial frequency of 18 cycles/º, which was strongly correlated with the VA improvement, and was also in line with the higher size effect observed for medium and high compared to low frequencies. These findings mainly observed in high spatial frequencies agree with previously results reported by Polat in patching resistant children [21], and adults [24]. The increase in CS led the AE to achieve similar values than FE, which has been found to have a positive impact on binocular vision in amblyopia [25,35,36]. Avram et al. [13] also reported an increase in CS in 5 anisometropic amblyopic patients treated with Amblyopia iNET, reporting a pretreatment range of CS from 1.35 to 1.65 log units and post-treatment range from 1.35 to 1.95 log units.

From a neurophysiological point of view, PL-based training causes changes in response strength or tuning of individual neurons in the early visual cortex, which leads to plasticity-mediated changes [37]. This higher response depends on the orientation trained during the vision therapy and can justify the increase in VA and CS. Likewise, this is a process that seems to involve the whole brain, from striate visual cortex (V1) to extrastriate visual cortex and other brain areas (V3a, V4, V5, face fusiform area, and lateral intraparietal cortex among others), and is mediated by attention, stimuli complexity and type of the task used [38,39]. This fact highlights the relevance of adjusting the stimuli characteristics to the level of difficulty that can be tolerated by the patient, as did in this study, since increasing visual demands during visual tasks will promote the improvement in visual abilities through the adaptation of the brain to the new difficulty [40]. 

Although this is a pilot study, there is one important strength that should highlighted, which is the long-term follow-up. Likewise, this study shows the progress of the two intervention groups, and the potential benefits of PL for subjects who did not improve enough with only glasses and patching or did not undergo patching adequately. However, there are some limitations that should be acknowledged. First, results are based on non-parametric statistics due to the small sample and there is no placebo group, which means that practice effect can have some impact on results [41], although there is enough published literature that support our results. Consequently, conclusions of this study should be considered by clinicians with caution but can be the starting point of future research with a more consistent design. Second, compliance is a significant problem in amblyopia treatment since compliance was poor (40.2% in the first month) and decreased with time. This value is lower than other values published in the literature, as for example the 88.6% ± 18.9% and the 88.4 ± 18.7%) from Iwata [42,43] and the at least 50% from Birch et al. [15] at three months. Poor adherence was probably because the videogames used in this study are not attractive enough for patients. By extension, future research should analyze how make PL videogames more interesting to children for improving the adherence to treatment. Third, although this study suggests that PL can be like patching for VA recovering, it should be pointed out that patching continues to be the gold standard. Therefore, PL might be an additional approach to optimize the treatment or an alternative when patching is not enough or there is poor compliance. 

## 5. Conclusions 

A combined therapy of PL-based visual training and patching seems to be effective for improving VA in children with amblyopia who did not recover vision with patching alone previously. Subjects with poor adherence to patching can also benefit from PL, although patching remains to be the gold standard. CS also improves with PL-based training in the AE, mainly for mid and high frequencies. This improvement allows the AE to achieve similar values than the FE, and can have some positive impact on binocular vision, although it should be further investigated along as how to improve compliance. The most relevant improvement in VA and CS seems to occur in the first month after treatment and remains stable until 18 months later, but with an increasing trend. Results obtained in the current vision therapy group were similar to patching but considering that older children were treated and all of them with a previous failure with patching alone. Furthermore, there were no differences by type of amblyopia, and no regressions were observed. In conclusion, PL-based vision therapy alone or in combination with patching seems to be a potential treatment for amblyopia in children who did not obtained total vision recovery with patching alone.

## Figures and Tables

**Table 1:** Descriptive characteristics of the study sample.

	PL Group Perceptual Learning Group (*n*= 14)	Patching Group Patching Group (*n* = 13)	*p*-Value
Age (mean ± SD years)	9.7 ± 1.4	5.0 ± 0.4	0.006 *
Gender (male/female)	6 M/8 F	6 M/7 F	0.863
Type of amblyopia	7 aniso/7 strab	7 aniso/6 strab	0.842
Patching regimen (mean ± SD hours)	4.1 ± 1.2	4.8 ± 0.5	0.440
Previous treatment	Yes 12/No 2	Yes 3/No 10	0.001 *
Amblyopic eye			
Sphere (mean ± SD) (D)	+4.98 ± 2.75	+4.61 ± 2.66	0.752
Cylinder (mean ± SD) (D)	−1.51 ± 1.19	−1.12 ± 1.27	0.338
Fellow eye			
Sphere (mean ± SD) (D)	+2.10 ± 2.14	+3.34 ± 2.92	0.342
Cylinder (mean ± SD) (D)	−0.25 ± 0.47	−0.60 ± 0.78	0.228

* Statistical significance *p* < 0.05; Aniso: Anisometropic amblyopia; Strab: Strabismic amblyopia.

**Table 2:** Visual acuity measures during the follow-up. PL: Perceptual learning group.

		**LogMAR VA FE**											
	**Baseline**	**1 month**	**3 months**		**6 months**			**9 months**	**12 months**			**18 months**	
PL Patching	0.02 ± 0.04	−0.01 ± 0.02	0.066 ^b^	−0.01 ± 0.05	0.180 ^c^	0.00 ± 0.00	0.317 ^d^	0.00 ± 0.01	0.317 ^e^	0.01 ± 0.02	0.317 ^f^	0.0 ± 0.01	0.317 ^g^
	0.06 ± 0.10	0.10 ± 0.10	0.180 ^b^	0.05 ± 0.10	0.317 ^c^	0.06 ± 0.07	0.317 ^d^	0.04 ± 0.07	0.655 ^e^	0.00 ± 0.01	0.317 ^f^	0.02 ± 0.04	0.999 ^g^
*p-value*	0.519	0.043 *^,a^		0.234 ^a^		0.083 ^a^		0.129 ^a^		0.639 ^a^		0.648 ^a^	
		**LogMAR VA AE**											
	**Baseline**	**1 month**	**3 months**		**6 months**			**9 months**	**12 months**			**18 months**	
PL Patching	0.45 ± 0.38	0.25 ± 0.26	0.001 ^b,^*	0.20 ± 0.28	0.063 ^c^	0.20 ± 0.24	0.262 ^d^	0.19 ± 0.22	0.063 ^e^	0.19 ± 0.23	0.273 ^f^	0.26 ± 0.37	0.109 ^g^
	0.30 ± 0.20	0.21 ± 0.19	0.018 ^b,^*	0.13 ± 0.11	0.655 ^c^	0.16 ± 0.14	0.317 ^d^	0.12 ± 0.15	0.655 ^e^	0.09 ± 0.10	0.180 ^f^	0.06 ± 0.06	0.285 ^g^
*p-value*	0.350 ^a^	0.877 ^a^		0.924 ^a^		0.840 ^a^		0.679 ^a^		0.755 ^a^			0.368 ^a^

* Statistical significance *p* < 0.05. A FE: Visual acuity of the fellow eye. VA AE: Visual acuity of the amblyopic eye. ^a^ = *p*-value for Mann–Whitney U test comparing PL Group with Patching Group. ^b^ = *p*-value for Wilcoxon Signed Rank test comparing 1 month follow-up with baseline. ^c^ = *p*-value for Wilcoxon Signed Rank test comparing 3 month follow-up with 1 month follow-up. ^d^ = *p*-value for Wilcoxon Signed Rank test comparing 6 month follow-up with 3 month follow-up. ^e^ = *p*-value for Wilcoxon Signed Rank test comparing 9 month follow-up with 6 month follow-up. ^f^ = *p*-value for Wilcoxon Signed Rank test comparing 12 month follow-up with 9 month follow-up. ^g^ = *p*-value for Wilcoxon Signed Rank test comparing 18 month follow-up with 12 month follow-up.
